# Variable Time-Step Physics Engine with Continuous Compliance Contact Model for Optimal Robotic Grinding Trajectory Planning †

**DOI:** 10.3390/s24051415

**Published:** 2024-02-22

**Authors:** Yongcan Zhou, Yang Pan, Junpeng Chen, Tianjian Lei

**Affiliations:** 1 Shenzhen Key Laboratory of Intelligent Robotics and Flexible Manufacturing Systems, Southern University of Science and Technology, Shenzhen 518055, China; zhouyc2021@mail.sustech.edu.cn (Y.Z.); chenjp2020@mail.sustech.edu.cn (J.C.); 12132267@mail.sustech.edu.cn (T.L.); 2 Shenzhen Key Laboratory of Biomimetic Robotics and Intelligent Systems, Department of Mechanical and Energy Engineering, Southern University of Science and Technology, Shenzhen 518055, China; 3 Guangdong Provincial Key Laboratory of Human-Augmentation and Rehabilitation Robotics in Universities, Southern University of Science and Technology, Shenzhen 518055, China

**Keywords:** physics engine, continuous contact model, compliant contact force, robotic grinding trajectory planning

## Abstract

In the transition from virtual environments to real-world applications, the role of physics engines is crucial for accurately emulating and representing systems. To address the prevalent issue of inaccurate simulations, this paper introduces a novel physics engine uniquely designed with a compliant contact model designed for robotic grinding. It features continuous and variable time-step simulations, emphasizing accurate contact force calculations during object collision. Firstly, the engine derives dynamic equations considering spring stiffness, damping coefficients, coefficients of restitution, and external forces. This facilitates the effective determination of dynamic parameters such as contact force, acceleration, velocity, and position throughout penetration processes continuously. Secondly, the approach utilizes effective inertia in developing the contact model, which is designed for multi-jointed robots through pose transformation. The proposed physics engine effectively captures energy conversion in scenarios with convex contact surface shapes through the application of spring dampers during collisions. Finally, the reliability of the contact solver in the simulation was verified through bouncing ball experiments and robotic grinding experiments under different coefficients of restitution. These experiments effectively recorded the continuous variations in parameters, such as contact force, verifying the integral stability of the system. In summary, this article advances physics engine technology beyond current geometrically constrained contact solutions, enhancing the accuracy of simulations and modeling in virtual environments. This is particularly significant in scenarios wherein there are constant changes in the outside world, such as robotic grinding tasks.

## 1. Introduction

With the development of robotic trajectory planning technology, a large number of robots [1] have reduced the need for manual labor. The grinding process requires more uncertain contact with the external workpiece, and the physical robot cannot determine whether the planned grinding contact force is appropriate directly. The physics engine [2] is the most important part of the simulator, used to process physical effects such as simulating rigid body motion, collision, gravity, and friction. Simulation needs to be carried out from the virtual environment, but the current physics engine is not accurate enough to pursue speed. If there are differences between the simulated contact force and the real contact force in the physics engine, the trajectory planning of the sim-to-real [3] process will be wrong.

Contact–impact events encompass intricate physical occurrences characterized by brief durations, substantial forces, rapid energy dissipation, and notable alterations in speed. Modeling these contact–impact phenomena is inherently demanding owing to their significance and complexity. Due to the importance and complexity of these contact–collision phenomena, modeling them is inherently demanding. These events involve numerous variables, such as the material properties of the contacting surfaces, the surface shape of the contacting bodies, and the constitutive relations. The abrupt velocity changes render the contact behavior typical of a nonlinear and non-smooth system.

In the realm of modeling methodology, various approaches have surfaced to describe contact response in the context of multibody dynamics. Broadly categorized, these approaches can be divided into two groups: methods based on “geometric constraints” and models relying on “contact forces” [4]. The “geometric constraint” methods assume that the colliding bodies remain sufficiently rigid to avoid deformation during the collision process. This approach is referred to as the non-smooth dynamic formulation [5]. Conversely, the “contact force”-based model relies on evaluating the spring–damper model as a function of penetration depth and relative velocity, known as compliance or penalty [6]. Unlike non-smooth formulations, the “contact force”-based model is considered compliant, as colliding objects can deform in the contact region. The “contact force”-based model, also known as continuous analysis, continuously evaluates the contact force as a function of penetration.

The linear complementarity problem (LCP) [7] stands as a widely adopted model for addressing impact in the realm of robotics, particularly within the framework of the “geometric constraint” method. In robotic simulations, complementarity is central, requiring either zero displacement or zero contact force. This simultaneous satisfaction implies that the product of two values is forever zero. Essentially, the LCP method is used to solve the geometric contact problem of two objects. The impenetrability of rigid bodies at the velocity level can be achieved by constraining motion or contact forces. Some well-known game physics engines, such as ODE [8], Pybullet [9], and Vortex [10], use equivalent replacements for friction, although inaccuracies arise due to the non-physical nature of the parameters [11]. This kind of replacement for the convenience of calculation is prone to errors in numerical integration, which further leads to the collapse of the system. RaiSim [12] achieved a great breakthrough in computer graphics by implementing a new iterative solution method through the commonly used Gaussian–Sedar (PGS) method. Drake employs its transition-aware solver TAMSI [13] to accomplish compliant contact with regulated friction. MuJoCo [14] greatly improves the constraint-solving method by relaxing parameters to formalize the problem. Due to its superior computing speed, MuJoCo is widely used in the reinforcement learning training of robots [15].

Adopting a compliant contact force model has proven helpful [16] in overcoming the limitations faced by traditional approaches. The core idea of the continuous compliant contact model is a spring damping model in which the surface is regularly filled with parameters related to the surface material coefficient [17]. According to the set components, the contact force can be achieved to bounce off, thereby preventing penetration at the force level. The determination of stiffness and deflection magnitudes for the spring–damper elements is carried out based on factors such as relative indentation, material properties, and surface geometries of the colliding bodies. After years of development, Flores et al. [18] and Gonthier et al. [19] proposed the continuous compliance contact model. Their model was continuously compared with real physical collisions, and experiments verified the reliability of the model. In the field of mechanical simulation, the method of calculating collision using compliant contact force is widely used due to its speed and ease of programming. At the same time, this model can perform dynamic calculations very well from the physical level, which is beneficial to the numerical stability of the entire system. Therefore, this model has been applied to many closed-source commercial programs, such as RecurDyn [20], EDEM [21], and ADAMS [22].

Unlike prevalent robotic simulators [23], which often rely on geometric constraints for solving the contact process, thereby confining rigid contact to an idealized state, this paper aims to address these limitations. Our proposed solution involves a variable time-step physics engine with a continuous compliance contact model [24]. Firstly, the bouncing ball experiment with different consumption coefficients was tested to verify the calculation effect of the contact model. Then, by comparing the stability experiments of our proposed method and advanced physics engines, the superiority of the variable time step was verified. Finally, grinding experiments were conducted to further verify the reliability of the conversion from virtual contact force to real. The innovation and primary contributions of this study are outlined below.

(1) Development of a Novel Physics Engine: Central to our innovation is the development of a new physics engine based on the continuous compliance contact model. The analytical solution of the model facilitates the dynamic adaptation of contact forces throughout the simulation, leading to variable time-step integrator behavior.
(2) Enhanced Collision Process Modeling: Our engine introduces a framework to model collision processes precisely. By adopting this advanced approach, we improve simulation-to-real conversion. This is achieved through careful calibration of stiffness and disturbance parameters, which is beneficial to enhancing the simulation accuracy of the entire simulation system.
(3) Redefining Conventional Methods: This study challenges the traditional geometric constraint-based methods, signaling a significant paradigm shift toward more realistic and robust simulations in robotics. By leveraging a swift and stable analytical solution for dynamic equations, our approach ensures rapid acquisition of feedback force.

The rest of this paper is organized as follows. We describe the overview of the whole system in Section 2. We focus on the principle of the contact solver in Section 3. We discuss the performance of ball experiments and robotic grinding experiments in Section 4. Finally, the conclusions of this study are summarized in Section 5.

## 2. Framework of the Variable Time-Step Physics Engine

**Figure 1:**
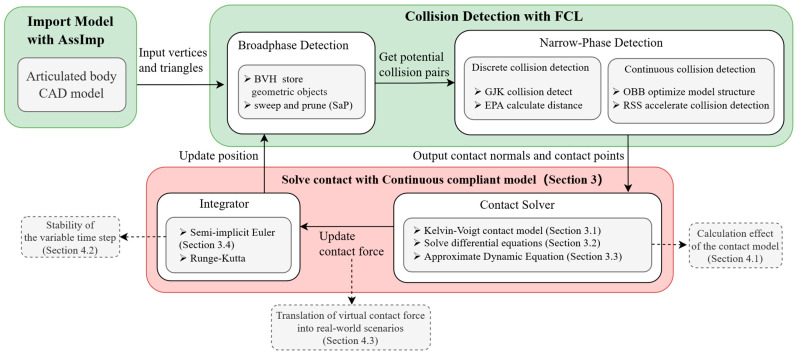
Framework of the variable time-step physics engine.

In this section, we propose the primary flow chart of the physics engine for robotics (referring to Figure 1). The components of the physics engine include the following: Firstly, Import the CAD model of the multi-jointed robot as a BVH model, which contains vertices and triangles. Then, the collision detection is calculated in FCL and the contact information is obtained, including whether there is a collision, the location of the collision point, and the collision normal vector. Contact forces are calculated in our proposed contact solver. Then, the next link position is further calculated in the integrator used. The dynamical calculation of the next time step is further updated and it continuously calculates the simulation.

Collision detection can provide the fundamental algorithms for contact solvers. Import the configuration of the robot and scene through XML files and save them as triangular patches for broad-phase detection. The data contain the model’s vertex and triangle needed for front-end display and collision detection, including vertex coordinates, triangle meshes, texture maps, and information about the object’s surface and material. This paper underscores the utilization of bounding volume hierarchies (BVHs), with a specific focus on oriented bounding boxes (OBBs), for the computation of separation distances across various robotic configurations [25]. In collision scenarios involving convex shapes, the Gilbert–Johnson–Keerthi (GJK) algorithm and the expanding polytope algorithm (EPA) stand out as the preferred methods. In continuous collision detection, the goal is to identify potential collisions along a known continuous path. This is achieved by sampling points along the path and performing periodic collision detection at these sampled locations. To enhance the efficiency of these tests, OBBs and rectangular swept spheres (RSSs) are employed as bounding volumes. The practical application of these algorithms is recommended to be carried out using the Flexible Collision Library (FCL) [26].

Utilizing collision detection outcomes, including contact position, normal vector, and penetration depth, this paper proposes a physics engine built upon a continuous compliance contact model. Section 3 is dedicated to detailing the contact solver. Data such as position, velocity, and contact forces obtained from the solver are reintegrated into the dynamical calculations. The robot’s position is updated by iterative application of semi-implicit Euler integration, and each new position is updated into the collision detection process.

## 3. Construction Contact Solver Based on Continuous Compliance Model

### 3.1. Kelvin–Voigt Contact Model

According to the spring-damping model theory, the contact model can be constructed initially [27]. Taking into account the material properties of the contact surface, the contact position and velocity, and the geometric characteristics of the impact surface, the Kelvin–Voigt model [28] is adopted. Then, the normal force is defined as

(1) $$F_{N}=D\dot{\delta }\left(t\right)+K\delta (t)$$

where $\dot{\delta }\left(t\right)$ is the penetration velocity about time t, $\delta \left(t\right)\;$ is the penetration about time t, and D is the viscous damping coefficient. The generalized stiffness parameter K relies on the material properties and the geometry of the contact surfaces. For contact between a sphere i and a plane surface body j, the generalized stiffness parameter depends upon the radius R of the sphere and the material properties of the contacting surfaces, and is expressed as [29]

(2) $$K=\frac{4}{3\left(\sigma_{i}+\sigma_{j}\right)}\sqrt{R_{i}}$$

in which the material parameters $\sigma_{i}$ and $\sigma_{j}$ are given by

$$\sigma_{l}=\frac{1-v_{l}^{2}}{E_{l}},\;(l=i,j)$$

The quantities $v_{l}$ and $E_{l}$ are, respectively, the Poisson’s ratio and Young’s modulus associated with each object. When it is the initial moment of collision, that is, time t  is 0, we can obtain the penetration $\delta \left(0\right)=0$. From the normal force balance, we can obtain the general dynamic equation as follows:

(3) $$F_{ext}=m\ddot{\delta }\left(t\right)+F_{N}=m\ddot{\delta }\left(t\right)+D\dot{\delta }\left(t\right)+K\delta \left(t\right)$$

where $F_{ext}$ means an external force, such as gravity or other external force acting on the object at the step time. Given the appropriate simulation step size, we can assume that the external force remains constant throughout the time step.

Solving the second-order inhomogeneous equation (Equation (3)) with initial conditions, $\delta \left(0\right)=0$ can obtain the penetration about time t:

(4) $$\delta \left(t\right)=e^{rt}\left[\frac{K\dot{\delta }\left(0\right)+rF_{\mathrm{ext}\;}}{K\omega }\sin\left(\omega t\right)-\frac{F_{ext}}{K}\cos\left(\omega t\right)\right]+\frac{F_{\mathrm{ext}\;}}{K}$$

where $r=-\frac{D}{2m}$, $\omega =\frac{\sqrt{4Km-D^{2}}}{2m}$.

Equation (4) serves as an analytical solution to the dynamical solution, allowing the physics engine to adjust the integration step with a variable time step during the contact process. This adaptive approach significantly enhances the stability of the system compared to geometric constraint solving.

The engine utilizes a combination of local error estimates and stability considerations, which together dictate the adjustment of the time step. The simulation step size is limited by collision detection and other dynamics calculation threads. The thresholds for these adjustments are determined in different scenarios based on empirical studies and theoretical models that predict the simulation’s behavior under variable conditions. Therefore, in order to ensure the reliability of the simulation, the simulation step size must be smaller than the minimum time period of other computing threads. By implementing this strategy, we aim to strike a balance between computational efficiency and the fidelity of the simulation outcomes, ensuring that the selected time step remain within bounds that preserve the integrity and reliability of the simulation’s dynamics.

### 3.2. Contact Force Calculation

After obtaining the position (Equation (4)) of the object during the collision process, further calculations are needed to obtain the contact force of the object within the integration step. Integrating the contact force $F_{N}$ (Equation (1)) within time Δt, we can obtain

(5) $$\int_{t_{0}}^{t_{1}}\;F_{N}dt=\int_{t_{0}}^{t_{1}}\;(D\dot{\delta }(t)+K\delta (t))dt\;\begin{array}{l} =\frac{\left(AK+DAr+DB\omega \right)\left[\omega \sin\left(\omega t\right)+rcos\left(\omega t\right)\right]e^{rt}}{r^{2}+\omega^{2}}\\ +\frac{\left(BK+DBr-DA\omega \right)\left[r\sin\left(\omega t\right)-\omega cos\left(\omega t\right)\right]e^{rt}}{r^{2}-\omega^{2}}+F_{ext}t\left|\begin{array}{c} t_{1}\\ t_{0} \end{array}\right. \end{array}$$

where $t_{0}$ and $t_{1}$ represent the initial and end moments of each contact. And $A=-F_{ext}/K$, B= $\left(K\dot{\delta }\left(0\right)-Kr\delta \left(0\right)+rF_{ext}\right)∕\omega K$.

In order to find the expression of the contact force model without the viscous damping coefficient ‘D’, we need to use energy conservation to further build the contact force model. The simplest way to quantify the energy loss during a contact event is to use the concept of coefficient of restitution, which can be evaluated from the balance of energy that occurs between the initial instant of contact $t^{\left(-\right)}$ and the final instant of contact $t^{\left(+\right)}$. Equation (3) shows that, since the motion process of spring damping is an analytical solution, the entire collision process time is known as

(6) $$t=\pi \sqrt{\frac{m}{K}}$$

By deriving Equation (4), the analytical solution of velocity can be obtained. Therefore, by incorporating Equation (6) into the analytical solution for velocity, we can obtain the velocity at the final collision moment. And we denote the velocity of the i-th object at the initial collision moment as $v_{i0}$ and at the final collision instant as $v_{i1}$.

(7) $$v_{i1}=-\dot{\delta_{0}}e^{-\xi \frac{\pi \omega }{\omega_{d}}}$$

where $\xi =\frac{D}{2m\omega }$, $\omega_{d}=\omega \sqrt{1-\xi^{2}}.$ According to the principle of energy conservation, the energy change in the collision process is

(8) $$∆E=\frac{1}{2}m_{i}v_{i0}^{2}-\frac{1}{2}m_{i}v_{i1}^{2}=\frac{1}{2}m_{i}v_{i0}^{2}\left(1-e^{-\xi \frac{2\pi \omega }{\omega_{d}}}\right)$$

Defined by the coefficient of restitution, we have $c_{r}=-{\dot{\delta }}^{+}/{\dot{\delta }}^{-}$. The energy variation within the collision process can be described as

(9) $$∆E=\frac{1}{2}m_{i}v_{i0}^{2}-\frac{1}{2}m_{i}v_{i1}^{2}=\frac{1}{2}m_{i}v_{i0}^{2}\left(1-c_{r}^{2}\right)$$

Solving Equations (8) and (9) yields the relationship between D and $c_{r}$. Subsequently, we can obtain the available collision contact force:

(10) $$F_{N}=-2\left|lnc_{r}\right|\sqrt{\frac{Km}{\pi^{2}+\ln^{2}c_{r}}}\dot{\delta }\left(t\right)+K\delta \left(t\right)$$

By utilizing the analytical solution from the contact model, the proposed physics engine can obtain the compliant contact force throughout the collision. This feature ensures the stability of simulation dynamics calculations and enables the simulator to capture dynamic changes in contact forces accurately. As a result, the simulator offers a realistic modeling of the robotics interactions with its environment, ensuring the reliability of the results.

### 3.3. Continuous Compliance Contact Model-Based Approximate Dynamic Equation

Hunt and Crossley [30] introduced a comprehensive formulation for contact force, which is expressed as $F_{N}=K\delta^{n}(t)+\lambda \delta^{m}(t){\dot{\delta }}^{q}(t)$. Among them, n, m, and q are all constants. By incorporating a damping term $\lambda \delta^{m}(t)$ before $\dot{\delta }(t)$, the Kelvin–Voigt model can effectively emulate energy dissipation during the contact–impact process. However, a challenge arises in the Kelvin–Voigt model, as the computed contact force persists even when no deformation occurs in the initial and separation phases of collision, deviating from realistic behavior. Hunt and Crossley’s research has gained widespread recognition and laid the groundwork for the development of numerous other methods for evaluating contact forces. Among them, the most worthy of our attention is novel continuous contact force model [31] with arbitrary indentation depth and velocity exponent using the approximate dynamic equation and system dynamic equation. The coefficient of the damping term $\lambda \delta^{m}(t)$ can be replaced by a constant. From references [31], we can obtain

(11) $$\left\{\begin{array}{c} m_{0}\ddot{\delta }\left(t\right)+C_{1}K\delta^{m}\left(t\right)=0\;Compression\;phase\\ m_{0}\ddot{\delta }\left(t\right)+C_{2}K\delta^{m}\left(t\right)=0\;Restitution\;phase \end{array}\right.$$

where the constant term $C_{1},C_{2}$ is

$$C_{1}=\frac{m+1}{n+1}{\left(\frac{m_{0}\left(n+1\right){\dot{\delta }}^{(-{)}^{2}}}{2K}\cdot \frac{c_{r}^{2}+c_{r}^{q}}{1+c_{r}^{q}}\right)}^{\frac{m+1}{n+1}}+\frac{\lambda }{K}\left(\frac{2}{2+q}\right){\left({\dot{\delta }}^{\left(-\right)}\right)}^{q}$$

$$C_{2}=\frac{m+1}{n+1}{\left(\frac{m_{0}(n+1){\dot{\delta }}^{(-{)}^{2}}}{2K}\cdot \frac{c_{r}^{2}+c_{r}^{q}}{1+c_{r}^{q}}\right)}^{\frac{m+1}{n+1}}-\frac{\lambda }{K}\left(\frac{2}{2+q}\right){\left(c_{r}{\dot{\delta }}^{\left(-\right)}\right)}^{q}$$

In order to make the mathematical form easier to understand, the penetration δ(t) in Equation (11) is replaced by y, and variables of time t are replaced by x. By converting the format, the equation can be converted into the following format:

(12) $$\ddot{y}\left(x\right)=Ay^{m}(x)$$

where $A=-C_{i}K/m_{0}$. According to the initial conditions, we have the following constant terms:

$$y\left(0\right)=0,y^{\prime }\left(0\right)={\dot{\delta }}^{\left(-\right)}$$

The equation is mathematically the Emden–Fowler Equation. The solution can be obtained by looking up the table in [32] as

(13) $$x=\left\{\begin{array}{ll} \pm \int {\left(2Ay^{m+1}/(m+1)+C_{1}\right)}^{-\frac{1}{2}}dy & \;\mathrm{if}\;m\neq -1,\\ \pm \int {\left(2A\ln\left|y\right|+C_{1}\right)}^{-\frac{1}{2}}dy & \;\mathrm{if}\;m=-1 \end{array}\right.$$

Equation (13) is the solution to Equation (12), which is also the solution to Equation (11) and obtains the advantages of the Hunt and Crossley contact model. This, in turn, makes the obtained contact force closer to the real force, which further improves the accuracy of the physics engine.

### 3.4. Semi-Implicit Euler Integrator

In the previous section, we calculated the acceleration of the objects at each moment. In order to further update the physics engine, we need to calculate the velocity and position of the object. The Runge–Kutta integral is orders of magnitude better than the semi-implicit Euler integral for smooth dynamics, but its advantage is lost in the presence of contact dynamics [23]. To speed up the calculation, we employ semi-implicit Euler integration. According to [33], by assuming that the integration step size within Δt is constant, we have

(14) $$\left\{\begin{array}{c} v_{n+1}=v_{n}+\Delta t{\cdot a}_{n}\\ p_{n+1}=p_{n}+∆t\;{\cdot v}_{n+1} \end{array}\right.$$

where the subscript n represents the nth moment calculated by the physics engine, and n+1 is the next calculation cycle after one time step corresponding to n, $v_{n+1}$ represents the velocity at moment after an integration time Δt, $a_{n}$ represents acceleration, $v_{n}$ represents the velocity at moment n-th. Similarly, $p_{n+1}$ represents the position of the link at the moment after an integration time Δt. It should be noted that, for the purpose of integration accuracy, the position integral in semi-implicit integration is used $v_{n+1}$.

By incorporating the acceleration into the semi-implicit Euler integration, we can successively update the velocity and position of the robot at each integration step. These updates are then refreshed into the physics engine and synchronized to the display thread. This process enables the achievement of a precise simulation step size. The above is the calculation process of the entire physics engine. The code implementation of the entire calculation process can be expressed as Algorithm 1.
**Algorithm 1** Contact Solver base Force Given parameters K and  CR
 while True do  Get position of collide object $\delta_{i}[t]$
  collide(A,B)//Collision detection for object A and B  if number of contact > 0   Compute $I^{c}\;,{\ddot{r}}_{c}\;,\;I^{eff}\;$
   for each contact i do    if contact is start     then ccd $p_{i}[t]\leftarrow p_{i}[t-1]$
    else if contact is end     then $t\leftarrow t_{c}$//modify time of contact    end if    Compute $a_{i}\left[t\right],\;v_{i}\left[t\right],\;p_{i}\left[t\right]\;$ with integrator    Compute $f_{i}\left[t\right]\;\leftarrow F_{i}\left[t\right]/∆t$
   end for  end if end while

## 4. Experiment and Discussion

In order to verify the reliability of the simulation system with the above contact model, we initially conducted a bouncing ball experiment with varying coefficients of restitution to validate the accuracy of the contact model calculations. Subsequently, we compared the stability of our proposed method with advanced physics engines through stability experiments, confirming the superiority of the variable time-step approach. Finally, grinding experiments were performed to further validate the reliability of translating virtual contact forces into real-world scenarios.

### 4.1. Bouncing Ball Experiment

Designing bouncing ball experiments to verify contact models is a very effective and conventional method. The classic bouncing ball experiment [28] was selected for its exemplary capacity to illustrate the effectiveness and precision of our contact model in handling dynamic events, a critical focus of our research. At the same time, in the future we will also conduct a comprehensive analysis of the importance of a wider range of experiments to more strongly substantiate our claims.

**Figure 2:**
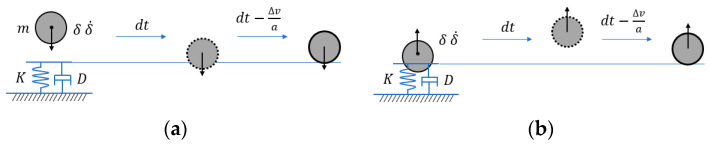
Continuous collision optimization initiation and end process: (**a**) The initiation moment of the collision; (**b**) The end moment of the collision.

As shown in Figure 2, a demonstration shows a sphere engaging in a perfect collision with the plane, starting from a height of 1.0 m. This sphere has a mass of 1.0 kg, a radius (R) measuring 0.1 m, and a moment of inertia of 0.1 kg·m^2^. The effective stiffness is 140 × 10^6^ N/m, determined using the formulation in Equation (1). The initiation of the sphere’s motion is solely attributed to the gravitational force acting in the downward (-y) direction from its starting position. Consequently, the ball undergoes free fall until it encounters the stationary and rigid ground, initiating a collision. Upon contact with the plane, a force is generated, prompting the sphere to rebound. This rebound imparts a specific height influenced by the coefficient of restitution.

During the penetration process, the colliding object will not be able to detect the contact surface if the step time is too long. Hence, the penetration process needs to be corrected, as shown in Figure 2a, using the movement process of the object before the contact collision to find a more accurate contact collision point. Similarly, at the end of penetration, as shown in Figure 2b, the contact collision process can be accurately modeled by correcting the collision time of objects according to the differential equation. This method is called ccd (continuous collision detection).

**Figure 3:**
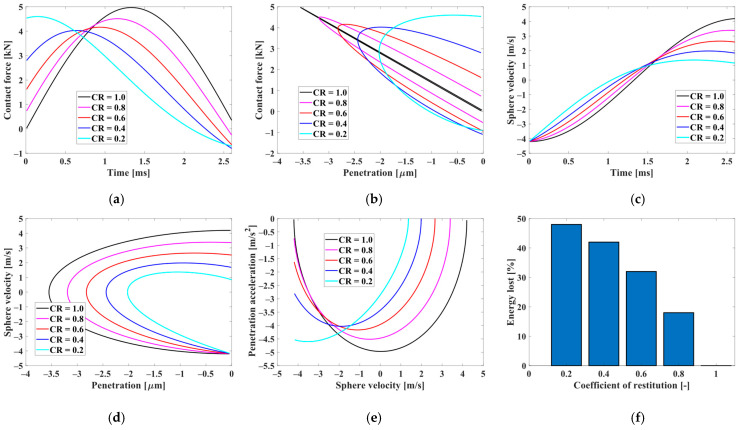
Modeling collisions between balls and surface using the Kelvin–Voigt contact force model at different coefficients of restitution (CR): (**a**) Contact force plotted against time; (**b**) Contact force plotted against penetration; (**c**) Sphere velocity plotted against time; (**d**) Sphere velocity plotted against penetration; (**e**) Penetration acceleration plotted against sphere velocity; (**f**) Energy dissipation percentage.

To investigate the influence of the coefficient of restitution (CR), a series of experiments on the contact dynamic response was conducted, which is depicted in Figure 3a–f. These graphical representations include time-dependent plots illustrating penetration, penetration velocity, and contact force observed during the initial contact. Additionally, Figure 3d illustrates the relationship between contact force and penetration. The analysis incorporates five different CR values: 0.2, 0.4, 0.6, 0.8, and 1.0. It is evident that, as the CR increases, the penetration of the sphere increases, accompanied by a rise in the maximum value of the contact force. This phenomenon results in a prolonged contact duration, and an enlarged hysteresis loop size, representing the energy dissipated during the penetration process. When the coefficient of restitution reaches 1, adhering to pure Hertz contact law, there is an absence of energy dissipation during penetration.

### 4.2. Performance of Calculation Stability

In a study conducted by [23], the stability of calculations in different physics engines for robotics was compared. The findings reveal a gradual onset of instability in the calculations as the step time increases. However, the proposed contact force calculation process, employing a continuous compliance contact force model, allows for the variable time step throughout the calculation process, ensuring the stability of the system. In order to detect the maximum simulation step time, we used the bouncing ball experiment when the coefficient of restitution was 1.0. By adjusting the time step, the experimental results can be obtained as shown in Table 1. It shows the maximum limit of each physics engine in adjusting the simulation step time. We can see that the proposed physics engine can achieve larger step size simulations by using the continuous compliance contact model, and the failure is due to the collision detection speed limit. Due to the advantage of using the contact model, the analytical solution calculated from Equation (4) furthermore ensures that our integrator ensures that the integration time step of the simulation is variable.

**Figure 4:**
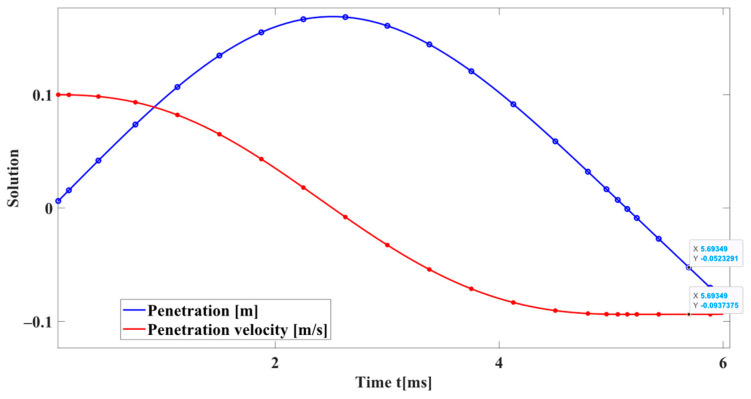
Solving a second-order nonlinear ordinary differential equation using the ODE78 method.

Observing Figure 4, it becomes evident that, when the coefficient of restitution reaches the unit, the energy within the contact model should be constant. When calculated by integration, a significant energy loss of 12% is observed using the kinetic energy change compared with ODE78 in Matlab. In cases involving multiple collisions, this cumulative loss can result in significant calculation errors, ultimately leading to highly inaccurate results.

### 4.3. Optimal Robotic Grinding Trajectory Planning

In production and manufacturing, optimized grinding trajectories can be achieved efficiently through simulation. However, it is necessary to have high virtual-to-reality reliability of contact force in the simulation. A grinding experiment is constructed to confirm the conversion effect of the proposed physics engine. The impact of contact force is then verified through roughness to further verify the reliability of the contact model. 

During grinding, the abrasive grains are subjected to the resistance of the deformation of the workpiece material and the friction between the abrasive grains and the workpiece surface, resulting in a grinding force. The grinding force can be decomposed based on the relative position of the workpiece and the grinding tool into a tangential component force, a normal component force, and an axial component force, with the normal component force generally being larger [35]. Furthermore, in the continuous contact model, the friction force can be derived from the normal force by formulas, which will be a focus of our future work. Therefore, normal force is currently selected for research, and other forces will be further studied in the future.

**Figure 5:**
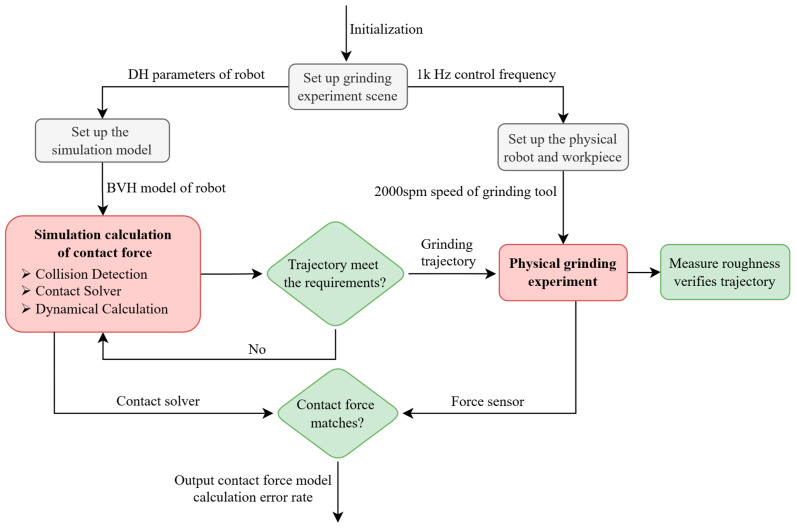
Simulation and physical grinding experimental process.

Figure 5 shows the entire grinding experiment process. Initialize and set the distance between the robot and the workpiece for the grinding experiment so that the simulation and physics experiment are consistent. Calculate the contact force of the grinding experiment in the robot simulation. If the designed trajectory meets the requirements, it will be sent to the actual robot for experiment. Verify that the trajectory is correct by measuring the surface roughness at the grinding location. At the same time, use the force sensor to measure the real contact force. And then compare the measured contact force with the calculated contact force, thereby assessing the effectiveness of contact models in the simulator.

**Figure 6:**
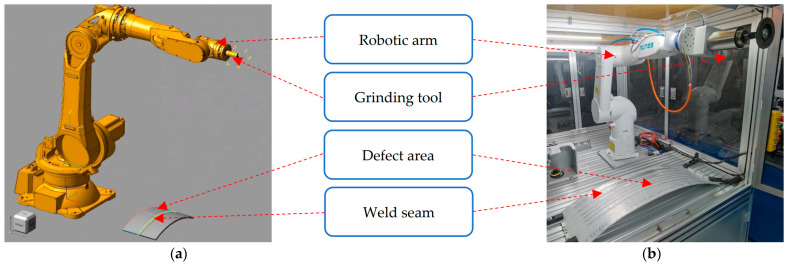
Comparison of experimental platforms: (**a**) simulation environment; (**b**) physical environment.

In this article, we use ESTUN’s ER10-900 industrial robotic arm for simulation and physical experiments, as shown in Figure 6.

The front-end of the developed robotic simulator uses the open source geometry engine Opencascade [36] as the display. The robotic dynamics algorithm then uses the open source library ARIS [37] for kinematics and dynamics solving. By starting the display thread, robot dynamics calculation thread, collision detection thread, and contact force solution thread at the same time, the grinding contact force under the determined stiffness coefficient and damping coefficient can be obtained through simulation.

In this article, the method parallel to the first selection curve is used to generate the surface grinding trajectory. The trajectory points are generated based on the surface position and normal vector of the curved surface. The advantage is that it can generate the required grinding trajectory for surfaces with convex shapes, greatly reducing manpower.

**Figure 7:**
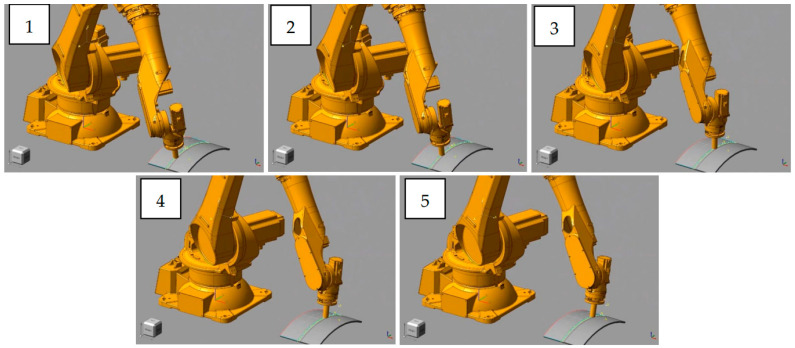
The grinding process by simulation. The numbers 1–5 represent the time sequence of the grinding process.

**Figure 8:**
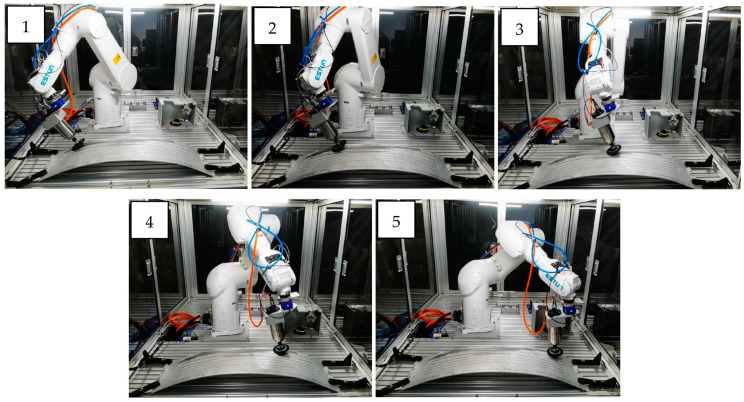
The grinding process by a real robot. The numbers 1–5 represent the time sequence of the grinding process.

Figure 7 and Figure 8 show the simulation and real robot grinding processes, respectively. The trajectory realized in the simulation is sent to the physical robot, and a control frequency of 1 kHz is used in the grinding workstation to ensure real-time performance. This high-frequency control ensures not only precision but also real-time responsiveness during the grinding operation. Due to the utilization of a position control both, a consistent trajectory is observed between the simulated robotic arm and the actual grinding experiment. This synchronization establishes a robust link between the simulated and real-world scenarios, providing a reliable foundation for further analysis and validation of our proposed methods. In the experiment, the choice of 5N was based on preliminary tests designed to study the system’s behavior under minimal stress conditions [38]. A force was chosen to be applied in the robotic grinding experiments, recognizing that this number may seem low compared to the higher forces typically used in industrial settings. However, because the grinding experiment can be repeated, the required grinding tasks can be completed in soft metal such as aluminum. Future work will extend the range of forces applied in our experiments to better understand the error behavior and its consequences at these higher force levels.

**Figure 9:**
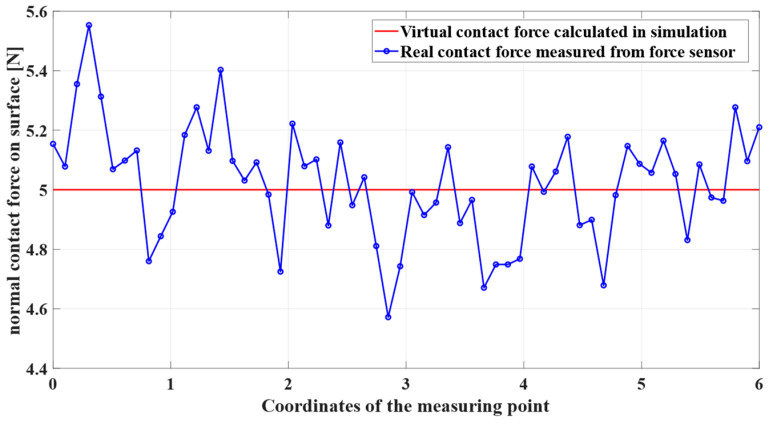
Grinding contact force of the surface.

By installing a force sensor between the end of the robotic arm and the grinding tool, we can measure the grinding contact force in real physical grinding experiments, as shown in Figure 9. Through twenty sets of experiments, we can compare simulated grinding experiments and real physical experiments. Changes in grinding force occur in physical experiments, which is a phenomenon that hardware experiments have to face. This phenomenon is primarily due to the random distribution of the abrasive grains on the grinding tool. These grains are polyhedral in shape, with each edge and corner acting as an individual cutting edge. Real grinding experiments require consideration of many factors, such as grinding workpiece surface, grinding speed, and grinding heat dissipation. Specifically, in this grinding experiment on soft metal aluminum, the irregularity of the weld and insufficient heat dissipation caused continuous fluctuations in the contact force. Despite these fluctuations, the results confirm that such changes do not affect the accuracy of continuous contact model force calculations in robot simulations, as demonstrated by experimental validation. Obtained through experimental data processing, the difference between the calculated force and the actual contact force was determined by calculating the average relative error, which showed a gap of 2.75%. Pre-calculating contact force in simulations can enhance the efficiency of conventional manufacturing processes to a certain extent.

Recognizing the importance of validating our model, we plan to undertake additional application experiments in the future. These experiments will aim to verify the model’s applicability to more complex, large-scale grinding operations, thereby enhancing its accuracy and reliability in industrial scenarios.

**Figure 10:**
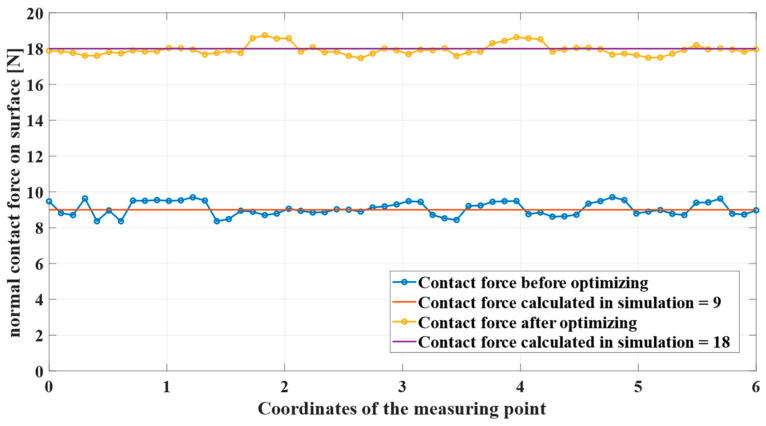
Grinding contact force of the surface before and after optimization.

As shown in Figure 10, they are the contact force of two different motion trajectories. It can be seen that the contact force was too small in the first experiment according to the grinding experience database. By adjusting the grinding strategy, the contact force during the entire grinding process can be more reliable with expectations and achieve better grinding results. Future efforts will focus on improving the contact model and physical conditioning. In addition, Section 4.2 demonstrates that variable time steps can be used for relatively fast and stable simulations. Therefore, precomputing and obtaining external forces from the simulation throughout the process facilitates grinding trajectory adjustment and helps improve efficiency in manufacturing.

**Figure 11:**
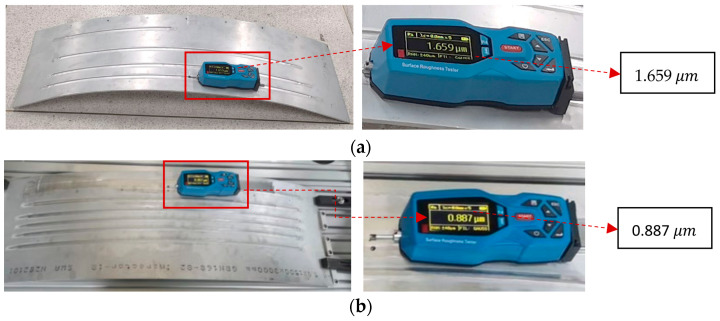
Grinding roughness measurement on the third same point: (**a**) Surface roughness before grinding; (**b**) Surface roughness after grinding.

**Figure 12:**
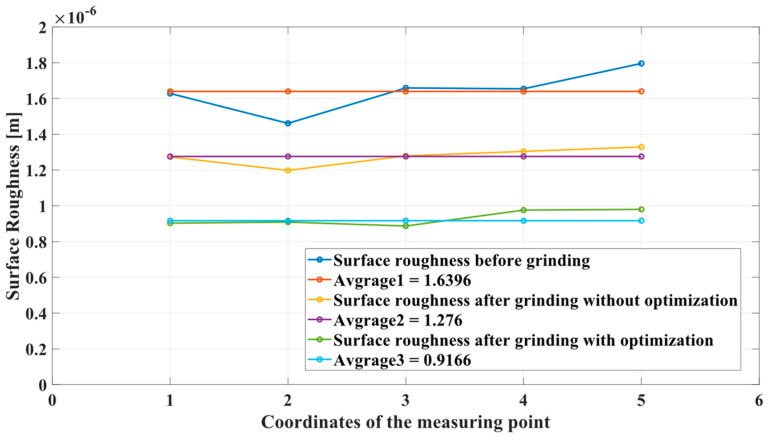
Roughness comparison of each point before and after grinding.

In order to observe the grinding effect from the side, we detect 10 sets of roughness on the grinding results. As shown in Figure 11, we measure and compare the roughness data of the third point before and after grinding. A total of 10 sets of roughness data were collected from the workpiece surface, revealing compliance with the grinding requirements. Figure 12 presents the roughness data for each point both before and after grinding. Notably, the initial roughness at each point appears to be relatively large, averaging 1.6396 [39]. Following the grinding process, a significant reduction in roughness at each point is observed, with minimal changes, averaging 0.9166. This corresponds to a 44.09% decrease in the average roughness value, affirming the effectiveness of the simulation’s strategy.

### 4.4. Discussion

The above experiments verified different aspects of the physics engine and the proposed features.

The bouncing ball experiment (Section 4.1) tested the contact model calculation effect through different coefficients of restitution. When the coefficient of restitution becomes one, it will become a completely elastic collision. The calculation results show that the contact force of the small ball can be accurately calculated as an analytical solution at different penetration depths or different moments.

Comparative experiments (Section 4.2) with different advanced physics engines verify the stability of the variable time step of the physics engine. Comparison of the previous literature [15] found that the maximum time step is only 16 milliseconds, which Mujoco can achieve. By employing a compliance contact force model and solving a second-order differential equation, the simulator achieves 128 ms as a max time step. Unlike the majority of existing robotic simulation engines that depend on geometric constraints to solve the contact process, resulting in hard contacts only existing in an ideal state, our approach employs the continuous compliance contact model with parallel springs and dampers, providing a stable representation of penetration.

We conducted grinding experiments (Section 4.3) on curved surfaces, discovering that optimal roughness during grinding [39] can be achieved by adjusting the contact force. The experimental findings reveal an average relative error of 2.75%, indicating a discrepancy between the virtual force and the actual contact force. In the future, as the contact model becomes more general, such as adding multiple points of contact, we will test experiments in more robotic scenarios. The adjustment not only enhances grinding simulation accuracy but also simplifies the verification process for grinding experiments.

Our results show that the physics engine equipped with the continuous compliance contact model can enable efficient simulations for robotics and in the future also help to effectively generate data for training artificial intelligence algorithms.

## 5. Conclusions

In this research, we propose a physics engine for robotic grinding, employing a continuous compliance contact model to calculate contact forces during virtual simulations. The contact force solver discussed in this paper relies entirely on the principles of spring damping, providing an analytical solution for contact forces in collision scenarios. This study features an illustration using the classic ball bouncing model, showing the simulator’s ability to calculate contact forces continuously. In contrast, contact solutions in game engines, relying on geometric constraints, often involve numerous non-physical parameters, leading to reduced accuracy and stability.

The proposed grinding simulator boasts several key advantages. Firstly, the contact model is grounded in the well-established classic spring–damping model in the mechanical field, incorporating penetration parameters for modeling, resulting in exceptional accuracy. The second advantage is that the simulator is able to obtain the contact forces in the analytical solution, allowing for a large degree of adjustment of the integration time step, thus ensuring more stable system performance. Additionally, analytical solutions to dynamic equations offer significant advantages in terms of computational efficiency and uniqueness. The availability of real-time feedback forces significantly enhances the overall stability of the system, further bolstering its robustness and reliability. Such advantages enable a more accurate simulation of complex grinding processes, aiding technicians in reducing manpower and material resources and ultimately improving experimental efficiency.

One constraint within the scope of this investigation pertains to the measurement of restitution coefficients, especially concerning diverse material surface properties. Consequently, there exists uncertainty in the generalizability of our findings to specific practical scenarios. Addressing this limitation necessitates additional research efforts that concentrate on constructing a comprehensive and dependable correlation model between the spring stiffness system and the damping coefficient. Another viable approach involves the exploration of swifter and more precise methods for identifying the coefficient of restitution, as such advancements enhance the accuracy and reliability of our findings in practical applications. Future work will continue to further optimize model storage and calculation, which will further ensure the speed of collision detection.

## Figures and Tables

**Table 1:** Comparison between the proposed and other advanced physics engines.

Physics Engine	Contact Solver	Max Timestep (ms)
Bullet [9]	MLCP	1/32
ODE [8]	LCP	1/4
PhysX [34]	LCP/PGS	2
Mujoco [14]	Newton/PGS/CG	16
Proposed	Continuous Compliance Model	128

## Data Availability

Data are contained within the article. If you need, you can find the source code at https://github.com/YongcanZhou/collision (accessed on 28 December 2023).
